# Orienting Toward Face‐Like Stimuli in Early Childhood

**DOI:** 10.1111/cdev.12441

**Published:** 2015-10-05

**Authors:** Punit Shah, Francesca Happé, Sophie Sowden, Richard Cook, Geoffrey Bird

**Affiliations:** ^1^ King's College London; ^2^ City University London; ^3^ University College London

## Abstract

Newborn infants orient preferentially toward face‐like or “protoface” stimuli and recent studies suggest similar reflexive orienting responses in adults. Little is known, however, about the operation of this mechanism in childhood. An attentional‐cueing procedure was therefore developed to investigate protoface orienting in early childhood. Consistent with the extant literature, 5‐ to 6‐year‐old children (*n *=* *25) exhibited orienting toward face‐like stimuli; they responded faster when target location was cued by the appearance of a protoface stimulus than when location was cued by matched control patterns. The potential of this procedure to investigate the development of typical and atypical social perception is discussed.

Faces contain a wealth of information crucial for social interaction (Adolphs, 1999), including identity (Bruce & Young, 1986), emotion (Darwin, 1872/1998), and personality traits (Willis & Todorov, 2006). Faces are also highly salient for humans; they are processed remarkably well from an early age (Crookes & McKone, 2009; de Heering, Houthuys, & Rossion, 2007), and may capture attention more effectively than other objects (see Palermo & Rhodes, 2007). Strikingly, even the youngest infants exhibit precocious face perception abilities. Despite their poor visual acuity, newborns preferentially orient to faces (Goren, Sarty, & Wu, 1975; Maurer & Young, 1983), look for longer at attractive than unattractive faces (Slater et al., 1998, 2000), and become sensitive to emotional expressions in the 1st year of their life (e.g., Leppänen, Moulson, Vogel‐Farley, & Nelson, 2007; Taylor‐Colls & Fearon, 2015).

Many facial‐orienting behaviors have been attributed to a mechanism tuned to a low‐spatial frequency configuration comprising three dark patches on a lighter background (hereafter “protoface”; Farroni et al., 2005; Morton & Johnson, 1991). Such a mechanism may serve a canalizing function, biasing input into newborns’ visual systems, thereby supporting the development of the cortical circuitry required for face‐processing and related social‐cognitive abilities (de Schonen & Mathivet, 1989; Farroni, Simion, Umiltà, & Barba, 1999; Johnson, 2005; Johnson et al., 2005). Experimental evidence suggests that orienting to face‐like stimuli may be underpinned by subcortical neural structures (Farroni et al., 1999; Simion, Valenza, Umiltà, & Barba, 1998; cf. Grossman & Johnson, 2014; though see Nelson, 2001).

It remains contentious whether orienting responses are specific to protoface stimuli per se, or whether they may be elicited by other types of top‐heavy patterns (Macchi Cassia, Turati, & Simion, 2004; Simion, Macchi Cassia, Turati, & Valenza, 2001; Turati, 2004). However, evidence for the specificity of protoface orienting has steadily accumulated. By employing various control stimuli (top‐heavy patterns, upside‐down, and negative protoface stimuli; see Figure 1B), convergent studies have shown that orienting responses are found selectively for upright protoface stimuli shown in positive contrast (Johnson, 2011; Johnson, Dziurawiec, Ellis, & Morton, 1991; Morton & Johnson, 1991; Valenza, Simion, Macchi Cassia, & Umiltà, 1996). The inability of the negative polarity protoface (white patches on a black background) to elicit orienting may reflect the fact that this arrangement does not resemble a face observed under natural lighting conditions (Farroni et al., 2005).

**Figure 1:**
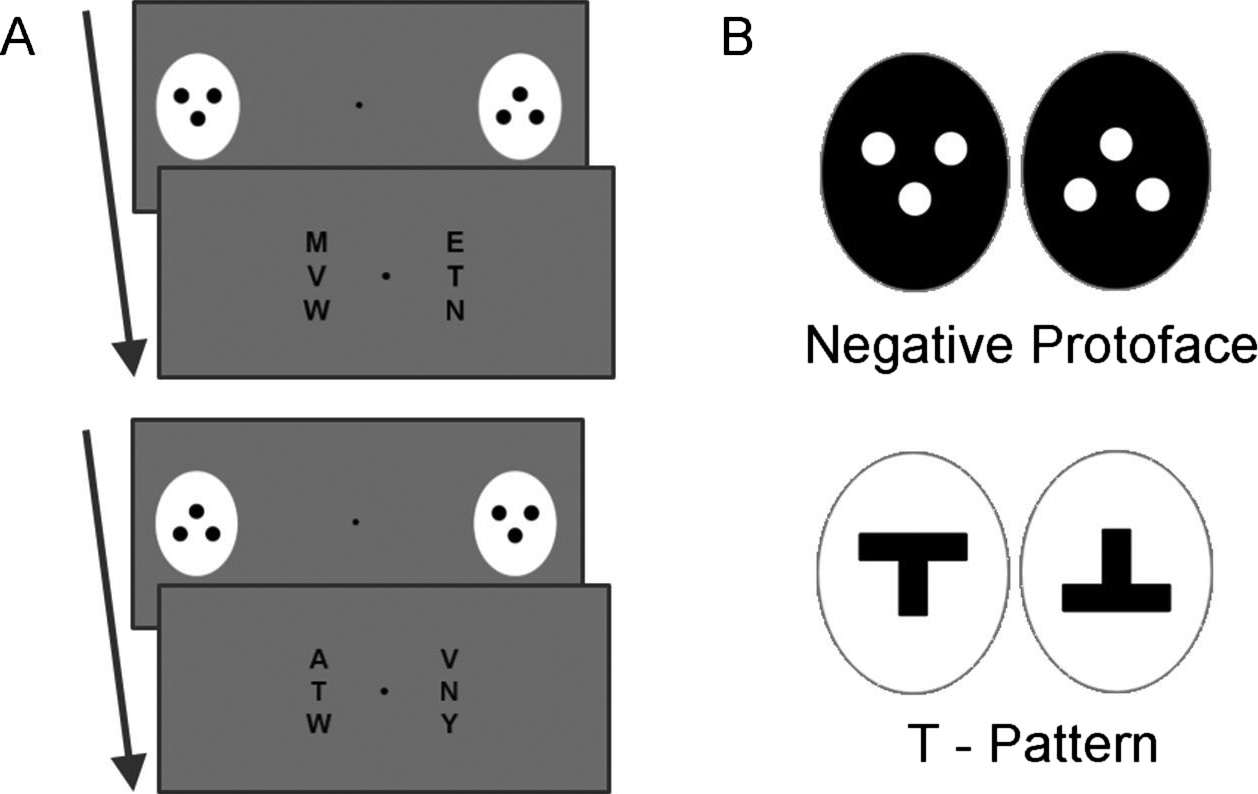
Stimuli and procedure. (A) Participants were tasked with indicating, as quickly and accurately as possible, which of the two letter arrays contained a target letter W. The arrays were separated by a central fixation cross. Before the arrays appeared on screen, an upright and upside‐down protoface were briefly presented in participants’ peripheral vision at left and right locations. The target letter then appeared either on the same (congruent trials) or opposite (incongruent trials) side of the display cued by the upright protoface stimulus. The difference in reaction times between congruent and incongruent trials (the congruency effect) served to index reflexive orienting. Control stimulus combinations (B) were presented using the same procedure.

An attentional bias for protoface stimuli has also been observed in adulthood. For example, having instructed participants to fixate rapidly on stimuli, Tomalski, Csibra, and Johnson (2009) found that adults were able to fixate the protoface faster than negative or upside‐down control patterns. Subsequent studies suggest that rapid protofacial fixation may be driven by the same mechanism responsible for preferential orienting during infancy (Gabay, Nestor, Dundas, & Behrmann, 2014; Stein, Peelen, & Sterzer, 2011; Tomalski & Johnson, 2012; Tomalski, Johnson, & Csibra, 2009). As participants were *explicitly* instructed to fixate on stimuli in these studies, this result could be due to either a reflexive orienting mechanism (i.e., exogenous attentional capture) or due to preferential orienting behavior (i.e., endogenous allocation of attention). To determine whether adults exhibit reflexive protoface orienting, Shah, Gaule, Bird, and Cook (2013) employed a cueing procedure: A target letter was presented at a random position within one of two arrays, arranged to the left and right of fixation. The onset of the letter arrays was briefly preceded by the protoface and an inverted control stimulus, presented simultaneously for 200 ms. Although participants were asked to ignore the patterns presented in the periphery, participants identified the location of the target faster when the protoface cued the correct array (congruent trials) than when it cued the incorrect array (incongruent trials). As the protoface and control patterns remained task irrelevant, these results indicate reflexive protofacial orienting in adults; their attention was captured by the protoface, even when it was unrelated to ongoing task performance.

In sum, there currently exists evidence for preferential orienting to protoface stimuli in newborn infants and reflexive protofacial orienting in adulthood. However, far less is known about orienting behavior during the intervening developmental stages. Specifically, it remains unclear whether protofacial orienting declines within the first months or years of life as originally proposed by Morton and Johnson (1991), to later re‐emerge in adulthood, or whether orienting toward face‐like stimuli is present throughout development (see also Mondloch et al., 1999). More generally, Lee, Anzures, Quinn, Pascalis, and Slater (2011) note that “while most of the recent exciting discoveries have been made with infants in all aspects of face processing, relatively limited knowledge has been gained about childhood except for the development of facial configural processing” (p. 771).

Changes in face‐processing ability sometimes follow nonlinear developmental trajectories (e.g., Chung & Thomson, 1995; Leonard, Karmiloff‐Smith, & Johnson, 2010; Mondloch, Dobson, Parsons, & Maurer, 2004; Mondloch, Le Grand, & Maurer, 2001; Short, Lee, Fu, & Mondloch, 2014). Moreover, there is evidence both for and against qualitative shifts in face perception ability during childhood (see Want, Pascalis, Coleman, & Blades, 2003), and a transient disruption during adolescence (Carey, Diamond, & Woods, 1980; Diamond, Carey, & Back, 1983; Thomas, De Bellis, Graham, & LaBar, 2007) possibly due to pubertal hormones (see Scherf, Behrmann, & Dahl, 2012). The development of face perception may also occur at a different rate to the maturational course of domain‐general mechanisms that mediate the perception of other objects (Pedelty, Levine, & Shevell, 1985; Scherf, Behrmann, Humphreys, & Luna, 2007; see also McKone, Crookes, Jeffery, & Dilks, 2012). However, considerably less work has sought to elucidate the role of facial orienting in the wider development of face processing, and whether protofacial orienting follows a nonlinear developmental trajectory. For example, orienting to face‐like stimuli might be strongest during critical periods of development, when input into the developing visual system will facilitate the emergence of perceptual expertise.

To begin addressing questions of this nature, it is first necessary to construct a developmentally appropriate behavioral task to index orienting to face‐like stimuli in younger and older children. This study therefore addresses whether the procedure developed by Shah et al. (2013) can be adapted to measure protoface orienting in early childhood. Specifically, we sought to determine whether 5‐ to 6‐year‐old children show robust and selective orienting to protoface stimuli.

## Method

### Participants

Twenty‐five children aged 5–6 years (*M *=* *5.28, *SD* = 0.46; 10 male) from a state‐funded elementary school in the United Kingdom (London) participated in the study in 2014. This age range was selected as it was likely to be the first period in early childhood where the majority of children would be able to complete the choice reaction time procedure. Due to the cosmopolitan nature of the school, the sample comprised a wide mix of nationalities and socioeconomic backgrounds. All children had normal vision and no known developmental, neurological, or psychiatric conditions. Both the children and their parents provided informed consent and the children received toy stickers for their participation. They were fully debriefed upon task completion, as were their parents and teachers. Ethical clearance was granted by the local ethics committee and the study was conducted in accordance with the ethical standards laid down in the 2008 (6th) Declaration of Helsinki.

### Materials and Procedure

On each trial two arrays of three letters (white, Arial font size 34) were presented 6° apart on either side of a black fixation cross on a gray background (128 on the decimal color scale; Figure 1). The letter arrays subtended approximately 3° × 1° of visual angle when viewed at a distance of 60 cm. Children were asked to detect a target letter (W) and indicate in which of the two letter arrays it appeared. The target letter was equally likely to occupy any of the six array positions, and distractor letters (Z, Y, X, V, T, N, M, L, K, H, F, E, or A) occupied the other five locations.

Immediately preceding the onset of the letter arrays, the protoface stimulus and an upside‐down protoface were presented for 200 ms, with the protoface cueing either the correct (congruent) or incorrect (incongruent) location of the target letter. Simultaneous presentation of the upside‐down pattern guarded against the possibility that cueing effects were due to low‐level features of the protoface (e.g., luminance, contrast, edge). To determine whether any cueing effect was specific to the protoface, or was due to a nonspecific feature such as its top‐heavy nature (Macchi Cassia et al., 2004; Turati, 2004), control stimuli consisting of a negative polarity protoface (three white patches on a black oval), or a nonface‐like top‐heavy pattern (T shape), replaced the protoface on control trials (Figure 1B). The protoface stimulus and control patterns subtended 4° × 3° of visual angle when viewed at a distance of 60 cm. Upright and upside‐down patterns were presented 12° apart. These were presented at an increased eccentricity with respect to the letter arrays so that cueing stimuli appeared in participants’ peripheral vision. Experimental programs were written in Matlab (MathWorks Inc., Natick, MA) using Psychtoolbox (Brainard, 1997; Pelli, 1997) and presented on a 15.6‐in. LCD monitor at 60‐Hz refresh rate.

Children were sat 60 cm from the display, and were instructed to disregard all peripheral stimuli while responding to the target letter as quickly and as accurately as possible. Ten practice trials were completed, with an opportunity to ask questions after five trials. The main experimental procedure comprised 180 trials, divided into five blocks of 30 trials, with equal numbers of positive polarity protoface, negative polarity protoface, and T‐pattern trials interleaved throughout the experiment. The complete procedure lasted approximately 8 min. Participants provided responses using both hands, using the left arrow (pressed with their left hand) and right arrow (pressed with their right hand) keys to indicate the array in which the target letter appeared. Reaction times (RTs) were measured from the onset of the letter arrays until a response was made and the difference in RT between congruent and incongruent trials (the congruency effect) served to index orienting.

## Results

Accuracy was almost at ceiling (*M *=* *94.96%, *SD* = 6.06%); therefore, mean RTs for each condition (Table 1) were calculated after exclusion of incorrect responses and RTs longer than 2 s. The number of excluded data points (12.7% of total) did not vary as a function of trial type as evidenced by one‐way analysis of variance (ANOVA), *F*(5, 144) = 0.51, *p *=* *.77, $\eta_{p}^{2}=.02$. Analyses were conducted on the resulting RT distributions, all of which were normally distributed (Kolmogorov–Smirnov tests, all *p*s > .65).

**Table 1:** Mean Reaction Times (s)

	Stimulus		
	Positive protoface	Negative protoface	T pattern
Congruent	1.360 (0.171)	1.419 (0.194)	1.401 (0.166)
Incongruent	1.436 (0.167)	1.390 (0.159)	1.380 (0.161)
Congruency effect	−0.076 (0.130)	0.029 (0.096)	0.021 (0.137)

Note.

Standard deviations are shown inside the parentheses.

**Figure 2:**
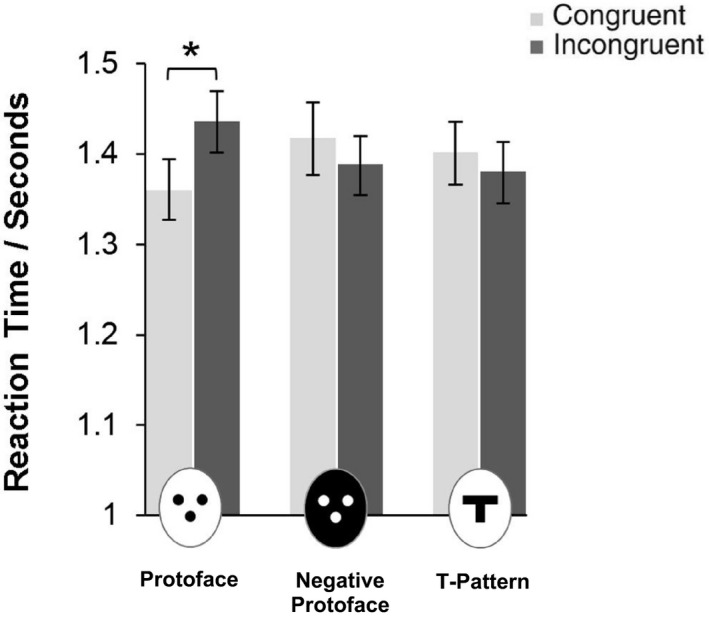
Experimental results—orienting to the protoface. Participants responded significantly faster to the target letter W when its location on the screen was cued correctly (congruent trials) rather than incorrectly (incongruent trials) by the protoface. This was indicative of orienting responses to the protoface stimulus. Control stimuli (negative protoface; T pattern) did not yield any significant congruency effects (Stimulus × Congruency interaction, *p *=* *.001). Error bars indicate ±1 *SE* of the mean. *p <* *.01, two‐tailed test.

Mean RTs (see Figure 2) were analyzed using ANOVA with within‐subjects’ factors of stimulus (positive polarity protoface; negative polarity protoface; T pattern) and congruency (congruent, incongruent). Neither the main effect of stimulus, *F*(2, 48) = 0.48, *p *=* *.64, $\eta_{p}^{2}=.02$, nor congruency, *F*(2, 48) = 0.25, *p *=* *.62, $\eta_{p}^{2}=.01$, was significant. However the interaction between Stimulus × Congruency factors was significant, *F*(2, 48) = 8.00, *p *=* *.001, $\eta_{p}^{2}=.25$. RTs were faster on congruent trials when the protoface had cued the correct side of the display than when cued by the negative protoface, *t*(24) = 2.29, *p = *.031, *d *=* *0.46, and the T pattern, *t*(24) = 1.90, *p = *.070, *d *=* *0.38. Equally, RTs were slower on incongruent trials when the protoface cued the incorrect side of the display than when cued by the negative protoface, *t*(24) = 2.54, *p = *.018, *d *=* *0.51, and the incongruent T pattern, *t*(24) = 2.84, *p = *.009, *d *=* *0.57.

Because the protoface, negative protoface, and T pattern are not matched in terms of low‐level features (e.g., luminance, contrast, edge), it is most appropriate to analyze performance within stimulus type as a function of congruency. Planned contrasts revealed that RTs were significantly faster when the protoface cued the correct rather than the incorrect side of the display when shown in positive polarity, *t*(24) = 2.94, *p = *.007, *d *=* *0.61. Crucially, however, congruency effects failed to reach significance for both the protoface shown in negative polarity, *t*(24) = 1.51, *p = *.15, *d *=* *0.30, and the control T pattern, *t*(24) = 0.77, *p = *.45, *d *=* *0.15. The difference in RTs between congruent and incongruent trials (the congruency effect) was significantly larger in response to the protoface than in response to the negative protoface, *t*(24) = 3.45, *p = *.002, *d *=* *0.69, and the T pattern, *t*(24) = 3.14, *p = *.004, *d *=* *0.63.

Orienting toward the protoface was not significantly different between left‐ (*n *=* *3) and right (*n *=* *22) handed children, *t*(23) = 0.01, *p = *.99, *d *=* *0.002, nor was there a significant difference between male and female participants, *t*(23) = 0.91, *p = *.37, *d *=* *0.38. While lengthy RTs were expected in a sample of this age (see Rueda et al., 2004), there was no association between mean RT across all conditions and the magnitude of orienting toward the protoface, *r *=* *.02, *p *=* *.92, nor was there any correlation between protoface orienting and age, *r *=* *.10, *p *=* *.62.

## Discussion

There is currently a paucity of methods to test facial orienting behavior in young children. The current study assessed whether a modified test of reflexive orienting—originally developed for adult participants—was suitable for use in 5‐ and 6‐year‐old children. The cueing procedure required children to identify the location of a target letter, when the protoface stimulus cued either the correct or the incorrect location. Children responded faster when the target location was cued by the protoface. Importantly, presentation of the protoface was entirely unrelated to the letter detection task, and the children were asked to disregard the briefly presented patterns. These results indicate that the protoface captures the attention of children, consistent with the reflexive orienting responses seen in adults (Shah et al., 2013). In accordance with previous research in infants and adults, orienting was not observed when the protoface was presented in negative polarity (see Johnson, Senju, & Tomalski, 2015). Contrary to the suggestion that orienting is elicited by top‐heavy patterns (Turati, 2004), a T‐shaped control pattern also failed to elicit the orienting responses. The selectivity of the cueing effect observed suggests that it may be mediated by the same mechanism responsible for preferential orienting in newborns (e.g., Farroni et al., 2005; Johnson, 2011).

### Typical Development of Social Perception

The present finding fits closely with the broader literature on developmental face processing, insofar as many basic aspects of face processing (sensitivity to facial attractiveness, gender, distinctiveness, race, and age) are evident in early childhood (see Lee et al., 2011). While it is impossible to draw strong inferences about neural mechanisms from behavioral findings, we suggest the possibility that reflexive orienting to face‐like stimuli in childhood is mediated in part by subcortical neural structures (amygdala, superior colliculus, and pulvinar), consistent with the interpretation of similar effects in the infant and adult literatures (see Johnson et al., 2015). These neural mechanisms have also been implicated in rapid orienting toward threatening stimuli, including threatening facial emotions (e.g., Morris, Öhman, & Dolan, 1999), and threat‐orienting effects are also evident during infancy and childhood (e.g., Leppänen et al., 2007; LoBue & DeLoache, 2010).

At present, it is uncertain how protofacial orienting changes with age or the extent to which it is influenced by genetic and environmental factors. This represents a potentially significant obstacle to explaining the factors contributing to the protracted development of face perception across early childhood and adolescence. This study represents an important step toward addressing research questions of this nature. Given the brevity of the procedure, it is amenable for use within (behavioral genetic; e.g., twin studies) longitudinal designs starting in early childhood. First, the use of this procedure will allow mapping of the (potentially nonlinear) developmental trajectory of protofacial orienting and its neurocognitive and genetic basis. Second, it may help clarify the contribution of orienting systems to the development across childhood and adolescence of the ability to recognize both identity and emotion. For example, it may enable a test of whether early individual differences in face orienting are associated with the perception of facial emotion and identity in childhood, adolescence, and in later life.

### Atypical Development of Social Perception

The use of the protoface stimulus has recently shown that, contrary to long‐standing theories of social impairments reported in autism spectrum disorder (ASD; e.g., Schultz, 2005), adults with ASD show robust orienting to protoface stimuli (Shah et al., 2013). Similarly, intact social orienting has been reported in young infants who are later diagnosed with ASD (Jones & Klin, 2013). Taken together, this indicates that ASD is not characterized by a lifelong impairment of social orienting and speaks against an innate social orienting impairment in ASD (see Johnson, 2014). Critically, however, little is known about the potentially detrimental consequences of an impaired social orienting mechanism during childhood. It is possible that children with ASD show a transient disruption in selectively orienting toward face‐like stimuli during a critical period of childhood development. This might explain, in part, reports of abnormal face perception or aberrant social cognition in individuals with autism. Given that orienting behaviors likely facilitate the development of various structures in the “social brain” (Johnson, 2005), the procedure described may prove useful for the study of atypical development of social cognition.

From studying individuals born with cataracts, it is known that being deprived of patterned visual input for just 2–6 months after birth can lead to devastating lifelong consequences on face‐recognition ability (Geldart, Mondloch, Maurer, de Schonen, & Brent, 2002; Le Grand, Mondloch, Maurer, & Brent, 2001). It is therefore possible that perturbations in orienting to face‐like patterns in childhood may also lead to face perception impairments in later life. The current procedure might therefore shed light on neurodevelopmental disorders that are characterized by impaired face recognition (see Johnson et al., 2005), such as developmental prosopagnosia.

### Limitations and Future Directions

Some caveats and limitations are worth noting. First, the cueing stimuli were designed to appear in children's periphery, and not to be fixated on. Peripheral processing seems likely given the viewing angles that were employed, the fact that stimuli were presented briefly, and that children were instructed to ignore peripheral stimuli. Nonetheless, in the absence of eye‐tracking data, it remains possible that some of the children attempted to fixate the cueing stimuli. It will therefore be of interest to administer the procedure in conjunction with eye tracking. Second, consistent with the previous literature (e.g., Johnson, 2005), we assume that orienting toward the protoface stimulus is driven by a subcortical neural mechanism and have therefore (tentatively) discussed our results in relation to the existing literature on this topic. Future work will greatly benefit from a direct investigation of the underlying neural basis of orienting behaviors in childhood. Finally, it is interesting to note that the orienting effect was larger and longer lasting than reported in adults (Shah et al., 2013). This is most likely to be due to procedural differences (i.e., fewer trials, differences in task difficulty), but at present there are no data that speak to this question. In combination with the orienting task that is suitable for use in adults (Shah et al., 2013), it is therefore hoped that the current procedure is employed within cross‐sequential or longitudinal designs, to investigate the developmental trajectory of protoface orienting from early childhood through to adulthood.

### Conclusion

Much is known about preferential orienting to face‐like stimuli in infancy and similar reflexive orienting effects have recently been reported in adulthood. However, relatively little is known about mechanisms supporting orienting behaviors in childhood, due in part to the lack of developmentally appropriate measures. This gap in the existing literature represents an obstacle to a comprehensive understanding of developmental face processing. We therefore describe an attentional‐cueing task with which we demonstrate robust orienting to face‐like stimuli in early childhood. This result accords with the extant literature on protoface orienting in infants and adults, and the broader literature on basic face‐processing mechanisms in childhood. The procedure described could be incorporated within sophisticated multivariate and longitudinal studies to further our understanding of developmental face processing.
